# Comparison of femoral tunnel position and knee function in anterior cruciate ligament reconstruction: a retrospective cohort study using measuring-fluoroscopy method versus bony marker method

**DOI:** 10.1186/s12891-024-07684-8

**Published:** 2024-07-23

**Authors:** Yan Dong, Yang Gao, Peng Cui, Yuanming He, Guke Yao

**Affiliations:** 1 Department of Orthopedics, Beijing Tongren Hospital, Capital Medical University, Beijing, China; 2 Department of Anesthesiology, Beijing Tongren Hospital, Capital Medical University, Beijing, China

**Keywords:** ACL rupture, Anatomical reconstruction, Bony landmarks, Measuring method, Apex, Cartilage, Fluoroscopy, Function

## Abstract

**Background:**

Previous studies have shown that surgical technique errors especially the wrong bone tunnel position are the primary reason for the failure of anterior cruciate ligament (ACL) reconstruction. In this study, we aimed to compare the femoral tunnel position and impact on knee function during the ACL reconstruction using measuring combined with fluoroscopy method and bony marker method for femoral tunnel localization.

**Methods:**

A retrospective cohort study of patients undergoing ACL reconstruction using the bony marker method or measuring combined with fluoroscopy for femoral tunnel localization was conducted between January 2015 and January 2020. A second arthroscopic exploration was performed more than 1 year after surgery. Data regarding patient demographics, the femoral tunnel position, results of the Lysholm score, the International Knee Documentation Committee (IKDC) score, KT-1000 side-to-side difference, pivot shift grade, and Lachman grade of the knee were collected.

**Results:**

A total of 119 patients were included in the final cohort. Of these, 42 cases were in the traditional method group, and 77 cases were in the measuring method group. The good tunnel position rate was 26.2% in the traditional method group and 81.8% in the measuring method group (*p* < 0.001). At the final follow-up, the Lysholm and IKDC scores were significantly greater in the measuring method group than the traditional method group (IKDC: 84.9 ± 8.4 vs. 79.6 ± 6.4, *p* = 0.0005; Lysholm: 88.8 ± 6.4 vs. 81.6 ± 6.4, *p* < 0.001). Lachman and pivot shift grades were significantly greater in the measuring method group (*p* = 0.01, *p* = 0008). The results of KT-1000 side-to-side differences were significantly better in the measuring method group compared with those in the traditional method group (*p* < 0.001).

**Conclusions:**

The combination of the measuring method and intraoperative fluoroscopy resulted in a concentrated tunnel position on the femoral side, a high rate of functional success, improved knee stability, and a low risk of tunnel deviation. This approach is particularly suitable for surgeons new to ACL reconstructive surgery.

## Background

Arthroscopic anterior cruciate ligament (ACL) reconstruction surgery is an effective treatment for ACL rupture. Previous studies have shown that surgical technique errors are the primary reason for the failure of ACL reconstruction [1–3]. Among these, the wrong bone tunnel position is one of the major factors leading to the failure of reconstructive surgery [1–3]. Previous literature has shown that about 50% of these failures are due to poor femoral tunnel position [4]. Anatomical studies have revealed the presence of bony landmarks on the medial wall of the lateral femoral condyle, such as the lateral intercondylar ridge and the lateral bifurcate ridge [5, 6]. Many clinicians have used these bony landmarks to locate the footprint of the ACL. However, Laverdiere et al*.* found that these bony landmarks are not always clearly identifiable intraoperatively [7]. van Eck et al*.* reported that the lateral intercondylar ridge could be observed in only 88% of patients and the bifurcate ridge in only 48% of patients [8]. Moloney et al*.* showed that by relying solely on bony landmarks for localization, more than 50% of the surgeons’ localization points would deviate from the original center of the footprint [9].

In order to find an objective position of the ACL on the medial wall of the femoral condyle and to ensure the satisfactory function of the knee, some positioning methods have been explored [4, 10–13]. Some surgeons refer to the remnant of the ACL for positioning during surgery [14, 15]. However, in some old ACL rupture cases, the soft tissue of the remnant in the footprint area has often been resorbed, making clear identification and accurate localization challenging [15, 16]. Another study reported that the femoral tunnel can be well-localized using the quadrant method based on intraoperative fluoroscopy [11]. Weiler et al*.* suggested that the posterior corner of the lateral meniscus can be used as a reference point for the localization of the femoral tunnel [17]. Some studies utilized self-developed drilling guides to assist in the localization and drilling of the femoral tunnel [18–20].

In clinics, these bony landmarks were not clearly displayed in many patients during surgery, which could affect the operator’s judgment of the location of the ACL. Thus, we need to find a new positioning method to complement the existing positioning techniques and achieve accurate positioning. According to anatomical studies, the posterior apex of the deep cartilage (ADC) and the center of the ACL femoral footprint was found to be in a stable position [11, 12, 21]. Hart et al*.* reported that at 90° of knee flexion, the center point of the ACL femoral footprint was an average of 3 (1–4) mm high and 12 (11–17) mm distant from the ADC [12]. Lee et al*.* suggested that the ADC could be a reference marker when reconstructing the ACL in a remnants-preserving manner [11]. The ADC position is stable and simple to reveal, and the ADC has a fixed positional correlation with the center point of the footprint.

The present study aimed to describe the outcomes of selecting the tunnel position by measuring combined with intraoperative fluoroscopy. In addition, a comparison was made between the different positions of the femoral tunnel and their effects on knee function.

Therefore, we hypothesized that this positional correlation could be used to select the location of the femoral tunnel using a ruler with ADC as the reference point. The position selected in such a way may be accurate and reproducible. However, considering the issues such as individual developmental differences, we also introduced a fluoroscopic method to correct the deficiencies of the measuring method and prevent large deviations in the position of positioning.

## Methods

### Study design and patients

This retrospective cohort study included consecutive patients with ACL rupture who underwent arthroscopic anatomic single-bundle ACL reconstruction at the Department of Orthopedics, Beijing Tongren Hospital, Capital Medical University, Beijing, China, between January 2015 and 2020. The inclusion criteria were as follows: (1) age 18–50 years; (2) ACL rupture in the knee joint, accompanied by medial or lateral meniscus injury; (3) graft an autogenous hamstring tendon; (4) patients treated by single-bundle anatomical reconstruction of ACL (through the anteromedial portal). The exclusion criteria were as follows: (1) reconstruction of multiple injured ligaments; (2) lesions in the bilateral knee joints; (3) reconstruction using other graft materials; (4) revision surgery and knee infection. This study was approved by the ethics committee of our hospital with the approval number TRKYEC2020-026.

Patients whose femoral tunnels were localized using the bony marker method comprised the traditional method group. Patients whose femoral tunnels were positioned by measuring ruler combined with intraoperative fluoroscopy were set up as the measuring method group. Patient charts were retrospectively reviewed for patients’ gender, age, side, mean time from injury to initial ACL surgery, mean time to second surgery, body mass index (BMI), tibial tunnel position, and graft diameter. International Knee Documentation Committee (IKDC) and Lysholm scores were obtained before the initial ACL reconstruction and secondary arthroscopy exploration surgery. The pivot shift and Lachman tests were performed under anesthesia to assess the stability of the knee joint before the initial ACL reconstruction surgery and secondary arthroscopy exploration surgery. Each patient underwent a KT-1000 arthrometer assessment of anterior tibial translation relative to the femur for laxity of the ACL, obtained before the secondary arthroscopy exploration.

### Surgical procedure

1. The patient is positioned in the supine position. An arthroscopic examination of the intra-articular situation is conducted to confirm the presence of an ACL rupture and to manage any associated intra-articular injuries.
2. The graft is excised and prepared for use. The graft is typically selected from the autologous hamstring tendon.
3. The positioning and preparation of the femoral tunnel.

**Fig. 1:**
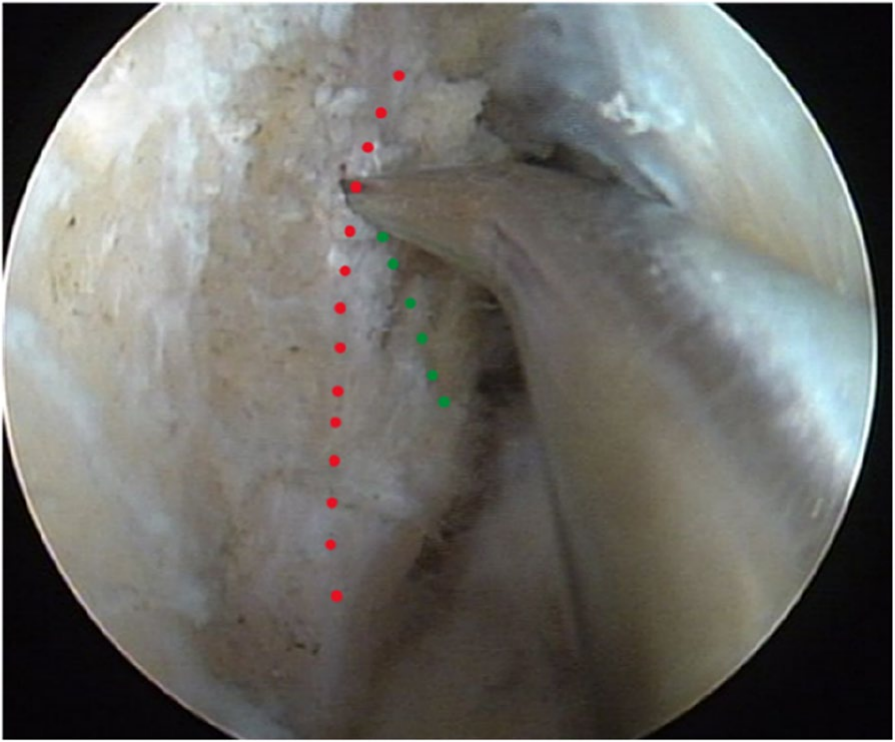
The femoral tunnel was positioned using the method of bony landmark (red dotted line: the lateral intercondylar ridge; green dotted line: the bifurcate ridge)

For the traditional method, the femoral tunnel center was selected at the intersection of the lateral intercondylar ridge and the lateral bifurcate ridge (Fig. 1).

**Fig. 2:**
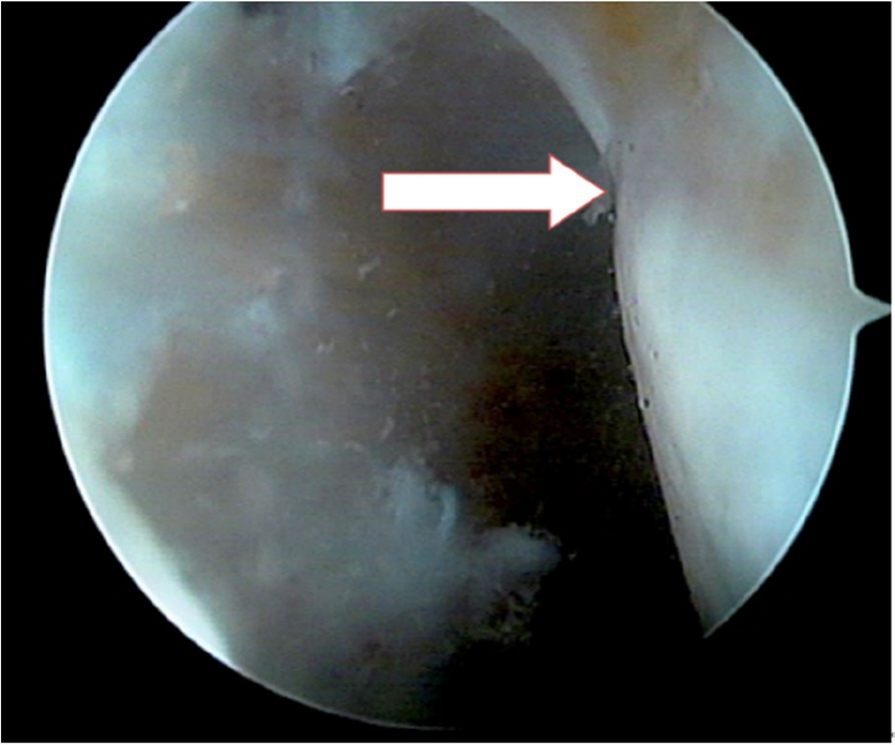
Access arthroscopic exploration through the intercondylar fossa to reveal the ADC (white arrow)

For the measuring method, the posterior cartilage margin of the femoral condyle was cleaned, and the ADC was revealed (Fig. 2).

**Fig. 3:**
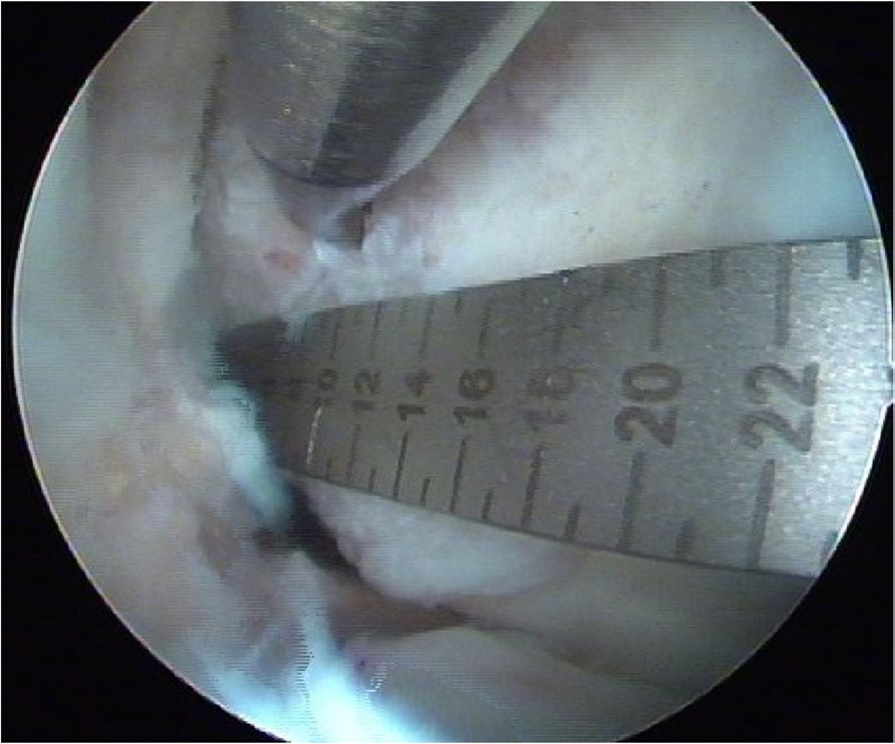
The 0-scale position was flushed with ADC, and a position 3 mm high and 12 mm distal to ADC was selected as the positioning point

The ruler was placed parallel to the long axis of the femur, with the 0-scale position flush with the ADC. A position 3 mm high and 12 mm distant from the ADC was selected as the positioning point, and the awl was used to position the femur at this location (Fig. 3).

**Fig. 4:**
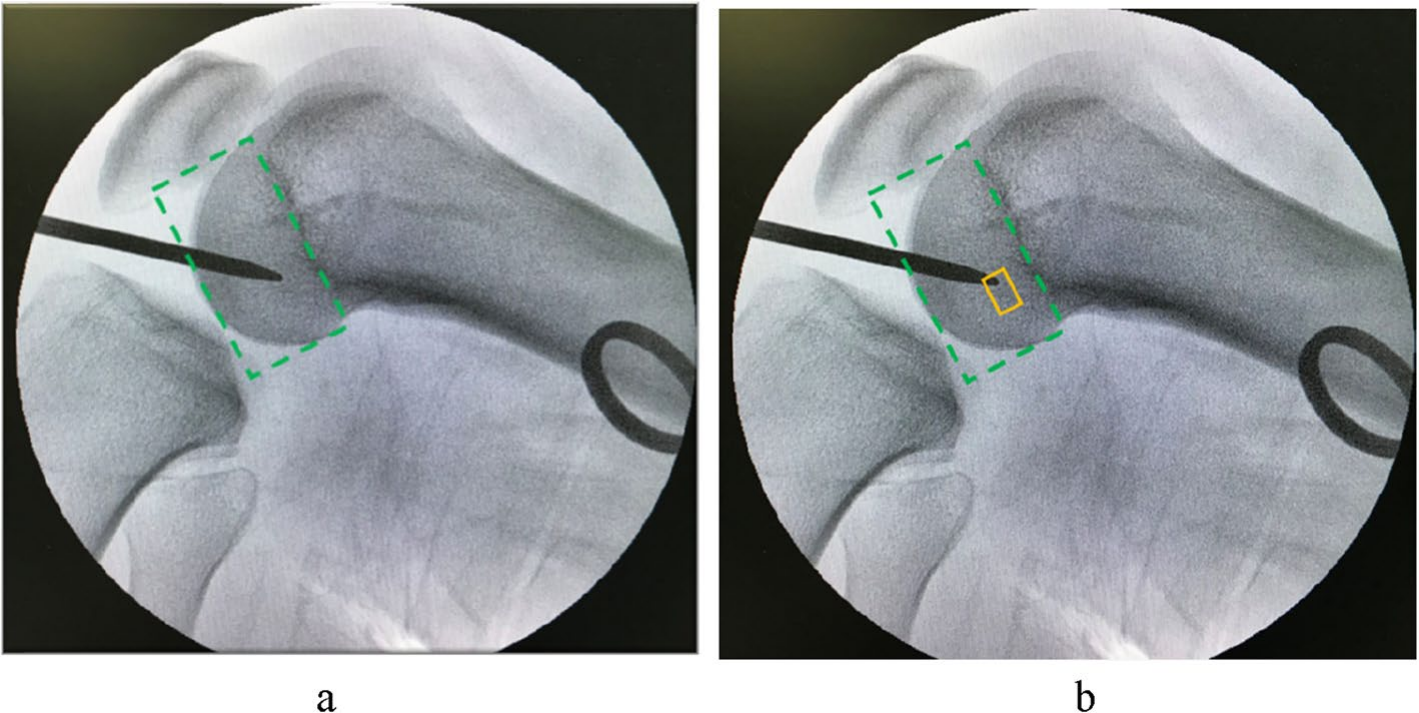
On the lateral image of the knee, a quadrant table was constructed to check if the localization point was within the normal zone (deep-shallow direction: 24–37%, high-low direction: 28–43%)

The lateral fluoroscopy of the knee was performed. On the lateral image of the knee, a quadrant table was constructed to check if the localization point was within the normal zone (Fig. 4). If it deviated from the normal area, the localization point was adjusted and confirmed fluoroscopically.

(4) Positioning and Preparation of the Tibial Tunnel

The location of the tibial tunnel can be determined by identifying the midpoint of the tibial stump of the ACL. In the event that the ACL stump has been resorbed and the location cannot be identified, the tibial tunnel can be positioned at the intersection of the extension line of the lateral meniscus and the vertical line of the midpoint of the intercondylar crest of the tibia.

(5) Implantation and fixation of the graft

The graft is retracted into the bone tunnel and the femoral side is secured with the Tightrope. An absorbable extrusion nail (absorbable screw: a mixture of lev polylactic acid and tricalcium phosphate, Arthrex Inc., USA) was employed to secure the graft on the tibial side.

### Postoperative care

On day 1 following ACL reconstruction, all patients began regular rehabilitation physiotherapy. The patient was discharged from the hospital and continued with their rehabilitation program until three months postoperatively. Following surgery, patients were required to wear a brace for a period of six weeks. The degree of knee mobility was observed to increase from 0–90 degrees in four weeks, with a return to the normal angle occurring between weeks six to eight. Knee weight-bearing: no weight-bearing for four weeks, partial weight-bearing for weeks five to six, and full weight-bearing after week six. The same rehabilitation team oversaw both the rehabilitation program and its execution. More than one year after the initial surgery, patients underwent a second arthroscopy to remove the internal fixation.

### Assessment of the tunnel position

A three-dimensional (3D) computed tomography (CT) reconstruction of the knee was performed after the surgery. The rectangular measuring frame was drawn on the medial view of the lateral femoral condyle, and the position of the tunnel on the medial wall was quantified.

**Fig. 5:**
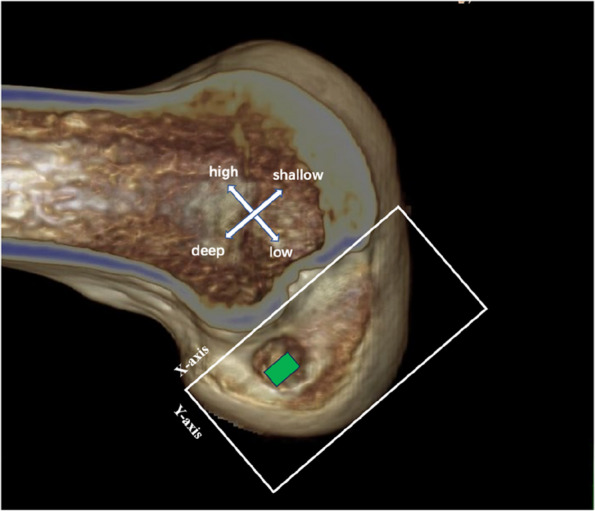
The normal range of tunnel centers on the femur (green box), X-axis deep-shallow direction: 24–-37%, Y-axis high-low direction: 28–43%

The normal range [22] of the tunnel positions at the medial wall of the lateral femoral condyle was 24–37% in the deep-shallow direction (X-axis) and 28–43% in the high-low direction (Y-axis) (Fig. 5). The bone tunnels were categorized into good and poor position groups based on whether the median site of the bone tunnel was in the normal range. The good position group included the bone tunnels in the range of 24–37% in the deep-shallow direction (X-axis) and in the range of 28–43% in the high-low direction (Y-axis). The poor position group included the bone tunnels beyond 24–37% in the deep-shallow direction (X-axis) or beyond 28–43% in the high-low direction (Y-axis).

### Statistical methods

The sample size was calculated using PASS 15.0 (NCSS, LLC. Kaysville, UT, USA) based on our preliminary retrospective analysis including 10 patients receiving traditional method. In these patients the incidence of good position is 25.0%. Considering a 30% increase in patients with measuring method, a sample size of 41 patients in each group was necessary to show a statistical difference with a power (1-β) of 80% and a two-sided-type I error of 5%.

Comparisons were made between patients who underwent primary ACL reconstruction with the traditional method *vs*. the measuring method. The SPSS 22.0 (IBM, NY, USA) software was used for statistical analysis. The age, body mass index (BMI), time from injury to initial surgery, the position of the femoral tunnel, KT-1000 side-to-side difference (SSD), and Lysholm and IKDC scores of patients before and after surgery in the two groups were compared using the t-test. The sex, side, pivot shift test results, and Lachman test results were compared using the chi-square (*χ*^2^) test. *P*-value < 0.05 was considered statistically significant.

## Results

A total of 136 patients who underwent ACL reconstruction were initially identified. 17 knees were excluded due to reconstruction of multiple injured ligaments (5 knees), revision surgery (2 knees), and loss to follow-up (10 knees). A total of 119 knees of 119 patients were included in the final cohort. The clinical characteristics of the patients are shown in Table 1.

**Table 1:** Baseline characteristics

	Traditional method group (*n* = 42)	Measuring method group (*n* = 77)	*P*-value
Left knee/right knee (cases)	26/16	40/37	0.29
Sex: male/female (cases)	32/10	67/10	0.13
Mean age ± standard deviation (years)	30.3 ± 5.2	31.3 ± 6.1	0.37
Mean time from injury to initial surgery (months)	9 ± 2.8	10 ± 3.1	> 0.05
Mean time to second surgery (months)	15.0 ± 3.0	16.0 ± 3.1	0.092
BMI	28.2 ± 3.8	29.3 ± 4.2	0.16
Tibial tunnel position (%)	41.5 ± 4.2	42.2 ± 3.1	0.24
Graft diameter (7/8/9 mm) (cases)	3/20/19	3/36/38	0.57

A total of 42 patients (mean age, 30.3 ± 5.2 years), including 32 males and 10 females, comprised the traditional method group. ACL reconstruction was performed in 26 left knees and 16 right knees. The average time from injury to the initial surgery was 9 ± 2.8 months, and that to the second arthroscopic procedure (removal of the tibial internal fixation) was 15.0 ± 3.0 months. The BMI of the patients was 28.2 ± 3.8, and the position of the tibial tunnel (anterior–posterior) was 41.5 ± 4.2%.

A total of 77 patients, including 67 males and 10 females, were in the measuring method group. The average age of the patients was 31.3 ± 6.1 years. The ACL reconstruction was performed in 40 left knees and 37 right knees. The average time from injury to the initial surgery was 10 ± 3.1 months, and the average time to the second surgery was 16.0 ± 3.1 months. The BMI of the patients was 29.3 ± 4.2, and the position of the tibial tunnel (anterior–posterior) was 42.2 ± 3.1%.

The gender, age, side, mean time from injury to initial ACL surgery, mean time to second surgery, BMI, tibial bone tunnel position, and graft diameter did not differ statistically significantly between the two groups (all *P* > 0.05) (Table 1).

The distribution of bone tunnel position in the X-axis was 5/29/8 (deep/normally/shallow) in the traditional method group compared to 9/68/0 in the measuring method group (*P* = 0.027). The distribution of bone tunnel position in the Y-axis was 27/13/2 (high/normal/low) in the traditional method group compared to 7/70/0 in the measuring method group (*P* < 0.001) (Table 2)*.*

**Table 2:** Distribution of tunnel position in the X-axis and Y-axis

	Traditional method group	Measuring method group	*P*-value
X-axis distribution (deep/normally/shallow)	5/29/8	9/68/0	0.027
Y-axis distribution (high/normal/low)	27/13/2	7/70/0	0.001

According to whether the center of the bone tunnel is located within the normal range, it is divided into good and poor positions. The rate of good tunnel position was better in the measuring method group (81.8%) than in the traditional method group (26.2%) (*P* < 0.001) (Table 3).

**Table 3:** Comparison of the two groups before the second ACL operation

	Traditional method group (*n* = 42)	Measuring method group(*n* = 77)	*P*-value
X-axis (%)	31.7 ± 6.3	26.1 ± 1.6	< 0.001
Y-axis (%)	22.1 ± 12.3	31.2 ± 3.1	< 0.001
Good positioned (cases)	11	63	< 0.001
Poor positioned (cases)	31	14	
Lysholm score (points)	81.6 ± 6.4	88.8 ± 6.4	< 0.001
IKDC score (points)	79.6 ± 6.4	84.9 ± 8.4	< 0.001
Pivot shift test (0 degree/1 degree/2 degrees/3 degrees, cases)	18/24/0/0	57/20/0/0	< 0.001
Lachman test (1 degree/2 degrees/3 degrees, cases)	24/16/2	60/17/0	0.01
KT-1000 side-to-side difference (mm)	4.1 ± 2.7	2.1 ± 1.8	< 0.001

The Lysholm score was 81.6 ± 6.4 for the traditional method group and 88.8 ± 6.4 for the measuring method group (*P* < 0.001). The IKDC score was 79.6 ± 6.4 for the traditional method group and 84.9 ± 8.4 for the measuring method group (*P* < 0.001) (Table 3)*.* At the final follow-up, 18/42 (42.9%) patients in the traditional method group had a positive Lachman compared to 17/77 (22.1%) in the measuring method group (*P* = 0.01) (Table 3)*.* A positive pivot shift was present at the final follow-up in 24/42 (57.1%) and 20/77 (26.0%) patients in the traditional method and measuring method groups, respectively (*P* = 0.0008) (Table 3)*.* At the final follow-up, KT-1000 side-to-side difference was 4.1 ± 2.7 mm in the traditional method group compared to 2.1 ± 1.8 mm in the measuring method group (*P* < 0.001) (Table 3)*.*

## Discussion

In the current study, compared to the traditional bony marker method, the measuring combined with fluoroscopy method for femoral tunnel position during ACL anatomical reconstruction resulted in a better femoral tunnel position and knee function. Thus, the measuring method may provide a supplement to the existing localization technique and thus, a new option for locating the femoral tunnel.

### The limitations of the bony marker method

Several studies have focused on the bony anatomical landmarks in ACL reconstruction. In an anatomic study, Ferretti et al*.* concluded that femoral tunnel positioning can rely on bony anatomic landmarks, such as the intercondylar and bifurcate ridges on the bone wall [5]. In choosing a technique to locate the center of the femoral tunnel, Steiner considered that the ideal method might accurately delineate the intercondylar and bifurcate ridges, but the process of ablating the tissue was time-consuming, and the ridges could be difficult to visualize [23]. Literature indicates that these bony landmarks are not always visible in all individuals and that intraoperative recognition is challenging [23, 24]. Furthermore, van Eck et al*.* reported that intercondylar ridges could be observed in 88% of patients, but bifurcation ridges can be observed in only 48% of patients [8]. These factors can affect the accurate localization of ACL insertion. Moloney et al*.* found that even when the bony landmarks are distinct, the location of the bone tunnel deviated from the central area of the ACL footprint in > 50% of patients when using anatomical markers alone for localization [9].

Our previous research found that the scattered location of the bone tunnel after the application of the bony marker localization [25]. Herein, we considered that the ligament stump is not recognizable in some old ACL rupture cases, and the bone crest and other markings are not clearly visible. In some cases of intercondylar fossa stenosis, the normal morphology of the bone wall was disrupted when intercondylar fossa plication was performed, resulting in unrecognizable bony markers, which in turn caused great difficulties for accurate localization. As a result, relying solely on these landmarks may cause the localization point to deviate from the bony tunnel’s normal range.

### Application of measuring method and fluoroscopy method

Bird et al*.* suggested that the ruler technique produced femoral tunnels similar to published radiographic criteria used for accurate tunnel placement [26]. Dong et al*.* reported a measuring method that positioned the tunnel in the center of the ACL femoral footprint [27]. Brown et al*.* [28] reported a measuring method that used a medial portal technique for single-bundle anatomical ACL reconstruction, and the technique had been suggested to be advantageous in that the position of the tunnel was related to the percentage distance along the ruler and was thus independent of the size of the knee.

Intraoperative fluoroscopy has been described as an accurate method to guide ACL femoral tunnel placement [29] and is reproducible and independent of different knee morphologies. Moloney et al*.* concluded that intraoperative fluoroscopy could successfully help the surgeon to position the femoral and tibial tunnels during ACL reconstruction and that consistency of positioning can be maintained [9]. Ahn et al*.* concluded that fluoroscopy-assisted positioning of the ACL anterior medial bundle could be placed more posteriorly than the conventional approach in ACL double-bundle anatomic reconstruction [30]. A comparison between positioning using the intra-articular bony marker method and positioning using the fluoroscopic method revealed that the position of the femoral tunnel was more accurate when positioning using the fluoroscopic method, especially in the high and low direction of the tunnel [31].

Although the use of intraoperative fluoroscopy can be an optimal solution for the accuracy of localization, using intraoperative fluoroscopy alone for localization might not locate the ideal position when selecting the localization point prior to fluoroscopy, especially for surgeons with limited surgical experience. If the position is not satisfactory, the positioning point should be adjusted according to the fluoroscopic position repeatedly. This action can result in bone wall destruction, compromising the integrity of the tip cone insertion position. The guide pin tends to slip during knee flexion, causing the actual position and the optimal fluoroscopic position to be inconsistent, creating the illusion of an exact position. Alternatively, if the bone wall is damaged or deficient, additional positioning sites must be chosen again, and the optimal positioning point may be lost. In a combination of measuring and fluoroscopic methods, the measuring method can ensure that the initial positioning is satisfactory, reducing the process of repeated positioner adjustments, while fluoroscopic positioning can further verify the accuracy of the position and avoid positioning errors, such as the complication of bursting the bone wall due to extreme posterior position.

In the current study, statistical analysis revealed that measuring combined with fluoroscopy provides a more accurate tunnel position. The ADC was used as the reference point, and the center of the ACL’s femoral footprint was 3 (1–4) mm high and 12 (11–17) mm distant from the ADC [11, 12, 27]. Because the anatomical position of the ADC is stable and easy to reveal surgically, we referred to ADC and chose a concentrated bone tunnel.

### Bone tunnel position and knee function

Some studies have shown that placing the graft in the femoral footprint brings it closer to the kinematics of the original ACL [32] 27). The femoral tunnel in the anatomic footprint also increases rotational stability without sacrificing anterior stability. Therefore, placement of the graft in the anatomical location would result in a stable knee joint [33], with biomechanical aspects playing a superior role in the control of rotational stability of the knee joint [34] and improved knee function. The present study also showed that after ACL anatomical reconstruction, the knee function scores improved in the measuring method group, along with the anteroposterior and rotational stability of the knee, which was related to the good femoral tunnel position.

These results suggested that the bone tunnel position was concentrated within the normal bone tunnel range in the measuring method group. Compared to the traditional method group, where the bone tunnel was scattered, the measuring method group had a better bone tunnel position and better performance in the Lysholm and IKDC scores of the knee, and the pivot shift test and Lachman test of the measuring method group showed better recovery of knee stability after surgery. Together, these findings indicated that restoring the anatomical femoral tunnel position can restore the biomechanical performance of ACL and obtain a stable knee performance.

In the present study, although we observed a mean difference of 5.3 points in the IKDC scores and 7.2 points in the Lysholm scores, which are considered statistically significant, they did not reach the minimum clinically important difference (MCID) as defined by the respective scales (9 points for IKDC and 12 points for Lysholm). However, we still consider these results to be of some value as they provide us with preliminary information on the effectiveness of our intervention and provide direction for future research. We suggest the following points be considered in future studies: expanding the sample size, extending the follow-up period, and combining other clinical indicators: (e.g., Pivot shift test, Lachman test, etc.) to comprehensively assess the effectiveness of the intervention.

Nevertheless, the present study has several limitations: (1) the sample size of this study is small; (2) it is inherently limited due to a retrospective design. Although this cohort of patients represents a consecutive series, the study is prone to selection bias as they were not randomized; (3) this is a short-term clinical observation, requiring additional studies to further explore other factors related to the functions.

## Conclusions

In conclusion, compared to the traditional method, the femoral tunnel was localized using measuring combined with fluoroscopy method; the position was accurate, the function of the knee could be improved, and the method was reliable. This approach is particularly suitable for surgeons new to ACL reconstructive surgery. Nonetheless, further studies are required to evaluate whether this method of femoral tunnel positioning has advantages over other approaches in terms of surgical outcomes.

## Data Availability

The datasets generated during the current study are not publicly available due to another article containing these data has not yet available published but are available from the corresponding author on reasonable request.
